# Identification of G protein subunit alpha i2 as a promising therapeutic target of hepatocellular carcinoma

**DOI:** 10.1038/s41419-023-05675-6

**Published:** 2023-02-20

**Authors:** Minbin Chen, Zhifei Li, Chengtao Gu, Hao Zheng, Yan Chen, Long Cheng

**Affiliations:** 1 Department of Radiotherapy and Oncology, Affiliated Kunshan Hospital of Jiangsu University, Suzhou, China; 2 Department of Interventional and Vascular surgery, Dushu Lake Hospital Affiliated to Soochow University, Medical Center of Soochow University, Suzhou, China; 3 General Surgery Department, The First Affiliated Hospital of Soochow University, Suzhou, China

**Keywords:** Targeted therapies, Liver cancer

## Abstract

Hepatocellular carcinoma (HCC) is a global health problem. Its incidence and mortality are increasing. Exploring novel therapeutic targets against HCC is important and urgent. We here explored the expression and potential function of Gαi2 (G protein subunit alpha i2) in HCC. The Cancer Genome Atlas Liver Hepatocellular Carcinoma (TCGA-LIHC) database shows that the number of *Gαi2* transcripts in HCC tissues is significantly higher than that in the normal liver tissues. Moreover, *Gαi2* overexpression in HCC correlates with poor prognosis of the patients. *Gαi2* mRNA and protein expression are also elevated in local HCC tissues and different human HCC cells. In patient-derived primary HCC cells and immortalized HepG2 cells, Gαi2 silencing (by targeted shRNA) or knockout (KO, by the dCas9-sgRNA method) largely suppressed cell proliferation and motility, while inducing cell cycle arrest and caspase-apoptosis activation. Moreover, Gαi2 silencing or KO-induced reactive oxygen species (ROS) production and oxidative injury in primary and HepG2 HCC cells. Whereas different antioxidants ameliorated Gαi2-shRNA-induced anti-HCC cell activity. Using a lentiviral construct, Gαi2 overexpression further augmented proliferation and motility of primary and immortalized HCC cells. Further studies revealed that the binding between the transcription factor early growth response zinc finger transcription factor 1 (EGR1) and *Gαi2* DNA promoter was significantly increased in HCC tissues and cells. In vivo, intratumoral injection of Gαi2 shRNA adeno-associated virus significantly hindered HCC xenograft growth in nude mice. Moreover, the growth of Gαi2-KO HCC xenografts in the nude mice was remarkably slow. Gαi2 depletion, oxidative injury, and apoptosis induction were detected in Gαi2-silenced or Gαi2-KO HCC xenografts. Together, overexpressed Gαi2 is required for HCC cell growth in vitro and in vivo, representing as a novel and promising diagnosis marker and therapeutic target of HCC.

## Introduction

Liver cancer causes about 800, 000 deaths each year globally [1, 2]. The incidence of hepatocellular carcinoma (HCC), the most common liver malignancy, has doubled in the past three decades in developed countries [1, 2]. Due to the increase in the incidence rate of non-alcoholic fatty liver disease (NAFLD), the number of HCC patients is expected to further increase, especially in developing counties [3, 4]. Most solid tumors have gradually declined with the development of current screening techniques and treatment measures, the incidence rate and mortality of HCC have yet been rising [3, 4]. Although surgery and local regional treatment are widely used worldwide, it is estimated that about 50 – 60% of HCC patients will eventually receive systematic treatment [3, 4]. Patients with recurrent and/or metastatic HCCs have extremely poor prognosis and survival is dismissal [5–7].

The multi-kinase inhibitor sorafenib has been utilized as the systemic treatment for unresectable or advanced HCC. Yet, sorafenib could only improve overall survival (OS) of the advanced HCC patients by approximately a few months [8]. Lenvatinib, also a multi-kinase inhibitor, can be utilized as a alternative to sorafenib for advanced HCC patients [9–12]. Whereas regorafenib, cabozantinib, and ramucirumab are appropriate supplements for sorafenib for the advanced HCC patients showing resistant to sorafenib [9–12]. Nivolumab and pembrolizumab, the PD-1/PD-L1 inhibitors, are being evaluated for the treatment of advanced HCC [9–12]. Even with the application of these molecularly targeted therapies, the prognosis and five-year overall survival of advanced HCC are still extremely poor. It is therefore urgent to explore novel therapeutic targets and signaling proteins essential for HCC progression, and to develop possible intervention against these targets.

The family of Gαi proteins, or G protein inhibitory α subunits, include three members: Gαi1, Gαi2 and Gαi3 [13, 14]. Gαi proteins are known to associate with G protein-coupled receptors (GPCR) and to block adenylate cyclase (AC) activation, thus reducing cellular cyclic AMP (cAMP) contents [13]. Our group and others have focused on the oncogenic roles of Gαi1 and Gαi3 in different human cancers, including glioma [15–17], cervical cancer [18] and osteosarcoma [19]. In these cancers, overexpressed Gαi1 and Gαi3 associated with receptor tyrosine kinases (RTKs) to mediate downstream Akt-mTOR cascade activation, thereby promoting cancer cell growth in vitro and in vivo [15–19].

Interestingly, several studies have implied a possible role of Gαi2 in carcinogenesis and cancer progression. Gαi2 upregulation in colitis-associated cancer was correlated with decreased relapse-free survival [20]. On the contrary, conditional Gαi2 knockdown in CD11c^+^ cells suppressed carcinogenesis of colitis-associated cancer [20]. Fu et al. discovered that overexpressed Gαi2 in epithelial ovarian cancer cells was important for cell growth [21]. Gαi2 silencing by a specific microRNA miRNA-222-3p remarkably inhibited epithelial ovarian cancer cell growth [21]. Importantly, Gαi2 could play an essential role in non-alcoholic steatohepatitis (NASH) progression [22] and its expression was significantly elevated in NASH patients’ liver tissues [22]. Moreover, conditional knockout of Gαi2 in hepatocytes prevented steatohepatitis development in mice [22]. These results implied a possible role of Gαi2 in HCC development. The results of the present study will show that overexpression of Gαi2 is important for HCC cell growth in vitro and in vivo.

## Materials and methods

### Reagents, chemicals and antibodies

Polybrene, puromycin, glutathione, EUK134, manganese tetrakis benzoic acid porphyrin (MnTBAP), antibiotics, serum, and medium were from Sigma-Aldrich (St. Louis, MO). Gαi1/2/3 antibodies and apoptosis-associated antibodies were described previously [23]. Anti-EGR1 antibody (ab194357) was from Abcam (Cambridge, UK). JC-1, CellROX, and other fluorescence dyes were provided by Dr. Ling [24].

### Cell culture

The immortalized HepG2 HCC cells and HL-7702 human hepatocytes, provided by the Cell Bank of Shanghai Institute of Science (Shanghai, China), were reported previously [25–27]. The patient-derived primary HCC cells, namely pHCC1, pHCC2, and pHCC3 (from three different written-consent patients), were described previously [25–27]. As reported [28], the primary human adult hepatocytes were obtained from the Cell Bank of Fudan University (Shanghai, China). These primary hepatocytes were derived from the liver of a partial hepatectomy patient with written-consent. Studies were conducted according to the principles expressed in the Declaration of Helsinki and national/international guidelines, and approved by the **t**he Ethic Committee of Soochow University (ID: 2021-BMR-014).

### Human tissues

The human HCC tumor tissues and the matched adjacent normal human liver tissues were from a total of twelve (*n* = 12) different primary HCC patients. All patients were enrolled at authors’ institutions and each provided written-informed consent for offering tissues for the research. Tissues were freshly obtained at the time of surgery and always stored in liquid nitrogen. The protocols were reviewed and approved by the Ethic Committee of Soochow University (ID: 2021-BMR-014). The protocols of immunohistochemistry (IHC) in human tissue slides were reported previously [16, 19].

### Short hairpin RNA (shRNA)

To knockdown Gαi2, the sequences encoding two different non-overlapping shRNAs, shGαi2-s1 and shGαi2-s2, were individually inserted into the lentiviral construct (GV248) (no GFP, Genechem, Shanghai, China), which was transduced to HEK-293 cells along with lentivirus-packing constructs (Genechem). The generated lentivirus was then added to cultured HCC cells or hepatocytes at multiplicity of infection of 10 (MOI = 10), and stable cells formed using puromycin selection medium (for 96 h). In the stable cells Gαi2 knockdown was verified at mRNA and protein levels. Control cells were stably transduced to the lentiviral non-sense control shRNA (sh-scr). For animal xenograft studies, the shGαi2-s1 or sh-scr sequence was sub-cloned into an adeno-associated virus (AAV) construct (AAV9, Genechem). The shRNA AAV was then generated and filtered using the attached protocols (Genechem). shRNA-induced silencing of early growth response zinc finger transcription factor 1 (EGR1) and stable cell selection was through the same protocol. The targeted sequences were: shEGR1-s1 and shEGR1-s2.

### Cas9-sgRNA-induced gene knockout (KO)

HCC cells were infected with the dCas9-expressing lentivirus (no GFP, Genechem, Shanghai, China), and single stable dCas9-expressing cells were formed after puromycin selection [29]. A total of three different CRISPR/dCas-9-Gαi2-KO constructs containing different small-guide RNAs (sgRNAs) against Gαi2 (“sgRNA1/2/3”) were synthesized and verified by Genechem. The construct, along with the lentivirus package constructs (Genechem), were co-transfected to HEK-293 cells, thereby generating lentivirus. The virus was filtered, enriched (at MOI = 15) and added to dCas9-expressing stable HCC cells. Cells were then distributed into 96-well plates and single stable cells (“koGαi2 sgRNA1/2/3”) were formed by puromycin-containing medium selection for additional 72 h, and Gαi2 KO verified by sequencing and Western blotting assays in the single stable colonies. The control primary HCC cells were stably transduced with a lenti-CRISPR/dCas-9 empty vector with non-sense sgRNA (“Cas9-C”).

### Gene overexpression

The Gαi2 cDNA ([NM_002070.4]) was inserted into the GV369 lentiviral construct, and the vector transfected to HEK-293 cells along with lentivirus-packing constructs (Genechem). Viruses were then filtered, enriched and added (at MOI = 15) to cultured HCC cells or hepatocytes. After selection by puromycin, two stable cell selections, oe-Gαi2-S1 and oe-Gαi2-S2, were formed. Overexpression of Gαi2 in the stable selections was verified at both mRNA and protein levels. Control cells were infected with empty vector-expressing lentivirus. Overexpression of EGR1 [NM_001964.3] was through the exact same procedure.

*Cellular functional studies*, including nuclear EdU (5-ethynyl-2’-deoxyuridine) staining assaying of cell proliferation, clonogenicity, CCK-8 assaying of cell viability, “Transwell” in vitro cell migration and “Matrigel Transwell” in vitro cell invasion assays, the caspase-3 activity assay, propidium iodide (PI)-flow cytometry assaying of cell cycle progression, nuclear TUNEL (terminal deoxynucleotidyl transferase dUTP nick end labeling) staining of cell apoptosis, Annexin V-PI flow cytometry assaying of cell apoptosis, Histone DNA ELISA, JC-1 (tetraethylbenzimidazolylcarbocyanine iodide) assaying of mitochondrial membrane potential were described in detail in other studies [17, 26, 27, 30–33]. Reactive oxygen species (ROS) assays, including CellROX staining, DCF-DA staining and single strand DNA (ssDNA) ELISA, were reported previously [24]. The superoxide dismutase (SOD) activity in fresh xenograft tissues was tested through a commercial SOD ELISA kit (Thermo-Fisher Invitrogen) according to the attached protocols. Western blotting and quantitative real-time PCR (qRT-PCR) have been described in early studies [23, 34, 35]. All mRNA primers were synthesized and verified by Genechem. Figure S1 listed the uncropped blotting images were listed in.

### Thiobarbituric acid reactive substance (TBAR) assaying of lipid peroxidation

Tissue or cellular lysates (35 μg per sample) were tested via a commercial TBAR kit (Cayman Chemical, MI), which quantified lipid peroxidation and measured malondialdehyde (MDA) contents colorimetrically at room temperature. TBAR optical density was examined at 545 nm with the reference of 600 nm. TBAR intensity was expressed in nmol per mg of total protein and was always normalized to that of control.

### *Gαi2* promoter luciferase activity assay

The predicted EGR1-binding site [36] was sub-cloned into a GV238 firefly luciferase vector [37] (Genechem). The described HCC cells were cultured at 60% confluence and were transfected with *Gαi2* promoter luciferase GV238 construct using Lipofectamine 3000 (Invitrogen, Shanghai, China). After 48 h, the firefly luciferase activity was measured by a Glo luciferase reporter assay kit (Genechem).

### Chromatin immunoprecipitation (ChIP)

ChIP assay protocols were described early [23]. Briefly, cell/tissue lysates were homogenized [38] and fragmented genomic DNA was achieved. Lysates were diluted and were immunoprecipitated (IP) with the anti-EGR1 antibody. EGR1-associated DNA was eluted. *Gαi2* DNA promoter sequence [36] was tested by quantitative PCR (qPCR) and its value was normalized.

### The xenograft studies

The nude mice, six-seven weeks old, 18.5-19.5 grams, half male half female, were provided by SLAC animal center (Shanghai, China). The xenograft model was described previously [30]. The primary human HCC cells, pHCC1 (at six million cells per mouse), were subcutaneously (*s.c*.) injected into the flanks of nude mice and pHCC1 xenografts were close to 100 mm^3^ after three weeks. Mice were then randomized assigned into two groups and ten mice in each group. Mice were subject to intratumoral injection of Gαi2-shRNA AAV (“aav-shGαi2”) or the same amount of scramble control shRNA AAV (“aav-sh-scr”). Tumor volumes, mice body weights, and estimated daily tumor growth were recorded as described. Alternatively, koGαi2 pHCC1 cells or the control Cas9-C pHCC1 cells (at ten million cells per mouse) were *s.c*. injected into the flanks of nude mice and pHCC1 xenografts measured after seven weeks. Gαi2 IHC staining in xenograft slides were described previously [18]. IHC intensity (“total gray”) was quantified through the Image J’s IHC Profiler. Its value was normalized to total cell number. Five random IHC images of each condition were included for the quantification. The nuclear TUNEL fluorescence staining protocols in xenograft slides were reported early [24]. The animal studies were approved by Institutional Animal Care and Use Committee and Ethics Committee of Soochow University.

### Statistical analyses

Data were always with normal distribution and were expressed as means ± standard deviation (SD).One-way analysis of variance (ANOVA) was performed for multiple group comparison, followed by Dunnett post hoc test using SPSS 23.0 (SPSS inc, Chicago, CA). The two-tailed unpaired student t test (Excel 2007) was utilized for the comparison of two groups. *P* values < 0.05 were considered statistically significant.

## Results

### Gαi2 overexpression in HCC

**Fig. 1:**
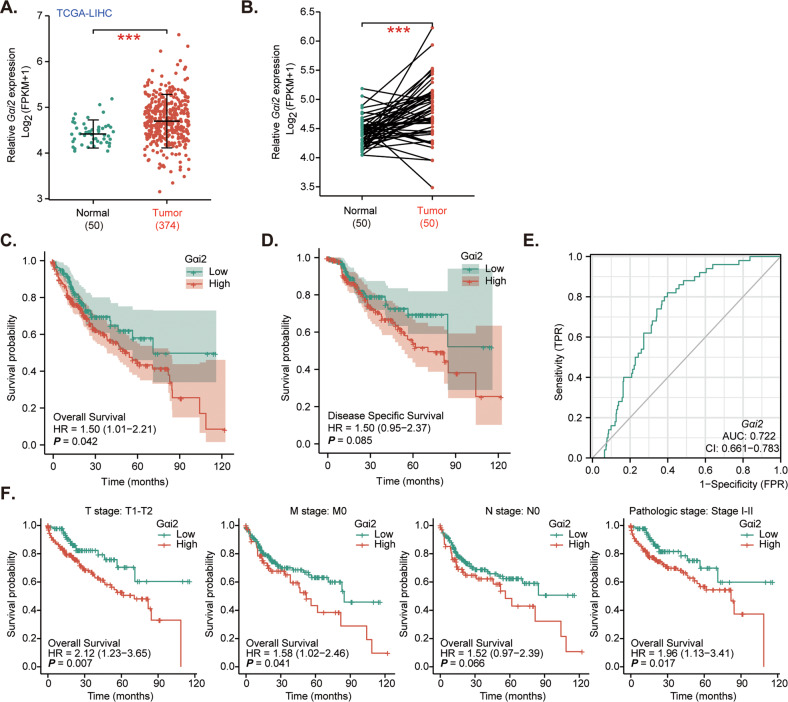
Gαi2 overexpression in HCC. The Cancer Genome Atlas Liver Hepatocellular Carcinoma (TCGA-LIHC) database shows *Gαi2* expression (RNA-Seq) in HCC tissues (“Tumor”, *n* = 374) and normal liver tissues (“Normal”, *n* = 50) (**A**). TCGA-LIHC shows *Gαi2* expression in HCC tissues (“Tumor”, *n* = 50) and matched adjacent normal liver tissues (“Normal”, *n* = 50) (**B**). The Kaplan–Meier Survival analyses of overall survival (**C**) and disease free survival (DSS, **D**) of *Gαi2*-low (in green) and *Gαi2*-high (in red) HCC patients from TCGA-LIHC. The receiver operating characteristic (ROC) curve showed the relationship between *Gαi2* overexpression and the potential predictive value on HCC patients’ overall survival (**E**). The subgroup analyses of *Gαi2* expression (RNA-Seq) and overall survival in the listed HCC patients were shown (**F**). “TPM” stands for transcripts per million. “AUC” stands for area under curve. “CI” stands for confidence interval. “HR” stands for hazard rate. “*TPR”* stands for true positive rate. “*FPR”* stands for false positive rate.*** *P* < 0.001 (**A**, **B**).

First, the bioinformatics analyses were carried out to examine *Gαi2* expression in HCC. The Cancer Genome Atlas Liver Hepatocellular Carcinoma (TCGA-LIHC) database was first consulted. A total of 374 HCC tissues (“Tumor”) and 50 normal liver tissues (“Normal”) were retrieved. As shown, the number of *Gαi2* mRNA transcripts in HCC tissues was significantly higher than that in the normal liver tissues (Fig. 1A). Further analyses showed that *Gαi2* mRNA expression in HCC tumor tissues (*n* = 50) was significantly higher than that in the matched adjacent normal liver tissues (*n* = 50) (Fig. 1B). Further analyzing TCGA-LIHC results revealed that the overall survival of *Gαi2*-high HCC patients was worse than that of *Gαi2*-low HCC patients (*P* = 0.042, Fig. 1C). Moreover, the *Gαi2*-high HCC patients tend to have worse disease specific survival (DSS) than that of the *Gαi2*-low HCC patients (*P* = 0.085, Fig. 1D). Area under curve (AUC) is an effective way to summarize and predict the overall diagnostic accuracy of a particular molecule in human cancer. The ROC curve in Fig. 1E evaluated the potential diagnostic value of *Gαi2* for HCC. With the AUC of 0.722, *Gαi2* overexpression should have an important value for potential HCC diagnosis. Further subgroup analyses showed that *Gαi2* overexpression in HCC was correlated with poor over survival in patients with T1-T2, M0, N0 and stage I-II HCC (Fig. 1F). These bioinformatics studies show *Gαi2* overexpression in HCC.

### Gαi2 is overexpressed in local human HCC tissues and different HCC cells

**Fig. 2:**
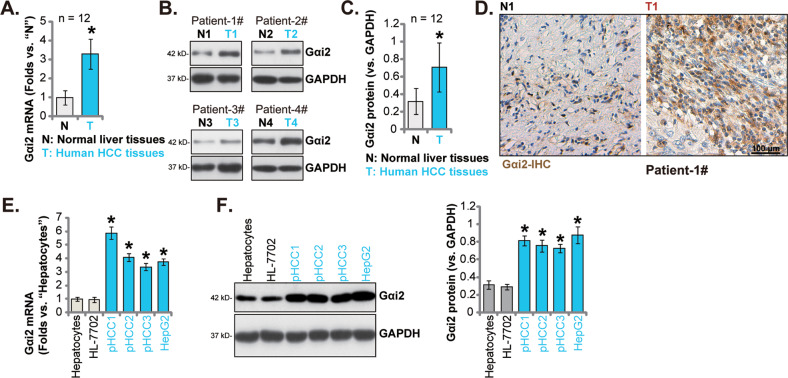
Gαi2 is overexpressed in local human HCC tissues and different HCC cells. Expression of *Gαi2* mRNA (**A**) and protein (**B**, **C**) in HCC tumor tissues (“T”) and matched adjacent normal liver tissues (“N”) of twelve local primary HCC patients was shown, with results quantified. The representative Gαi2 IHC images of HCC tumor tissue (“T”) and matched adjacent tissue (“N”) of Patient-1 were shown (**D**). *Gαi2* mRNA (**E**) and protein (**F**) expression in patient-derived primary HCC cells (“pHCC1/pHCC2/pHCC3”, derived from three different patients), immortalized HepG2 cells, HL-7702 hepatocytes or the primary human adult hepatocytes (“Hepatocytes”) was shown, with results quantified. The data were presented as mean ± standard deviation (SD).*P < 0.05 vs. “N” tissues/“Hepatocytes”. Scale bar= 100 μm.

To support the bioinformatics results, we tested Gαi2 expression in HCC tissues of local patients administrated at our institution. A total of twelve HCC tumor tissues (“T”) and matched adjacent normal liver tissues (“N”) were measured. As shown the *Gαi2* mRNA levels were significantly elevated in the HCC tumor tissues, where its expression was relatively low in the adjacent tissues (Fig. 2A). Gαi2 protein expression was upregulated in HCC tumor tissues of four different primary patients (Patient-1#/-2#/-3#/-4#), and relatively low Gαi2 protein expression detected in HCC-surrounding tissues (Fig. 2B). After combining Gαi2 protein blotting data of all 12 pairs of tissues, we showed that Gαi2 protein was significantly elevated in HCC tissues (Fig. 2C). IHC staining results, Fig. 2D, further confirmed Gαi2 protein upregulation in HCC tissues slides of Patient-1#. Next we tested Gαi2 expression in different HCC cells, including primary HCC cells derived from three patients, pHCC1, pHCC2 and pHCC3, as well as immortalized HepG2 cells. As shown, *Gαi2* mRNA expression in the primary human hepatocytes and immortalized HL-7702 hepatocytes was significantly lower than that in the primary (pHCC1/2/3) and immortalized (HepG2) HCC cells (Fig. 2E). Moreover, Gαi2 protein was upregulated as well in the primary and immortalized HCC cells (Fig. 2F). These results clearly show that Gαi2 is overexpressed in local human HCC tissues and various HCC cells.

### Gαi2 silencing/KO inhibits HCC cell progression in vitro

**Fig. 3:**
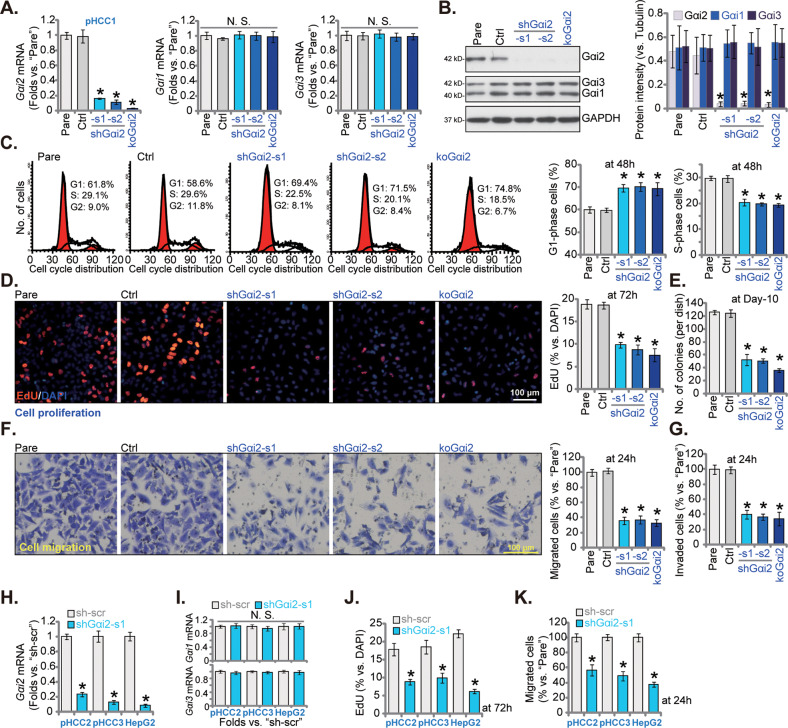
Gαi2 depletion inhibits HCC cell progression in vitro. The exact same amount of viable pHCC1 primary cells with applied genetic modifications on Gαi2, including shRNA-induced knockdown and dCas9/sgRNA1-induced knockout, were maintained in complete medium, expression of *Gαi1/Gαi2/Gαi3* mRNAs and listed proteins were tested by qRT-PCR (**A**) and Western blotting (**B**) assays, respectively. Cells were further cultivated for applied time periods, cell cycle progression, cell proliferation, colony formation, in vitro cell migration and in vitro cell invasion were examined by PI-flow cytometry (**C**), nuclear EdU staining (**D**), clonogenicity (**E**), “Transwell” (**F**) and “Matrigel Transwell” (**G**) assays, respectively, with results quantified and normalized; The control cells (“Ctrl”) were stably transduced with scramble control shRNA (sh-scr) plus lenti-CRISPR/dCas9-puro construct. The exact same amount of viable pHCC2 and pHCC3 primary cells, immortalized HepG2 cells, expressing shGαi2-s1 or sh-scr, were maintained in complete medium, and expression of *Gαi1/Gαi2/Gαi3* mRNAs was tested (**H** and **I**); Cells were further cultivated for applied time periods, cell proliferation (**J**) and in vitro cell migration (**K**) were tested similarly, with results quantified and normalized. “Pare” stands for the parental control cells. Data were presented as mean ± standard deviation (SD, *n* = 5). *P < 0.05 versus “Pare”/“sh-scr” cells. “N. S.” stands for non-statistical difference (*P* > 0.05). Experiments were repeated three times with similar results obtained. Scale bar= 100 μm.

To explore the potential effect of Gαi2 on HCC cells, pHCC1 primary cells were infected with lentiviral particles containing Gαi2 shRNA (shGαi2-s1 or shGαi2-s2, with different sequences). Stable cells were formed after selection using puromycin. Alternatively, the Gαi2 sgRNA1-expressing lenti-CRISPR/dCas9-KO-puro construct was transduced to dCas9-expressing pHCC1 cells, and stable Gαi2 KO cells (“koGαi2”) formed following puro selection and Gαi2 KO verification. The control pHCC1 cells (“Ctrl”) were stably transduced with scramble control shRNA (“sh-scr”) plus lenti-CRISPR/dCas9-puro construct. qRT-PCR assay results showed that *Gαi2* mRNA levels were substantially reduced in shGαi2-s1/2-expressing pHCC1 cells and koGαi2 (sgRNA1) pHCC1 cells (Fig. 3A), where *Gαi1* and *Gα3* mRNA expression was not significantly altered (Fig. 3A). Moreover, the applied shRNA strategy and koGαi2 (sgRNA1) resulted in remarkable Gαi2 protein downregulation in pHCC1 cells (Fig. 3B), without changing Gαi1 and Gα3 protein expression (Fig. 3B).

We next explored the functional consequence of Gαi2 silencing/KO in pHCC1 cells. PI-FACS cell cycle studies revealed that Gαi2 silencing/KO (sgRNA1) resulted in G1-S arrest (Fig. 3C). Following Gαi2 shRNA/KO (sgRNA1), G1 phase pHCC1 cells were increased but S-phase cells were decreased (Fig. 3C). Further experimental results showed that Gαi2 silencing/KO (sgRNA1) largely inhibited pHCC1 cell proliferation and significantly decreased EdU-positive nuclei ratio (Fig. 3D). Importantly, the number of viable pHCC1 cell colonies was robustly decreased following Gαi2 shRNA or KO (sgRNA1) (Fig. 3E). pHCC1 in vitro cell migration and invasion were tested by “Transwell” (Fig. 3F) and “Matrigel Transwell” (Fig. 3G) assays separately, and results showed that following Gαi2 silencing/KO (sgRNA1), pHCC1 cell motility was largely inhibited (Fig. 3F, G).

Two other CRISPR/dCas-9-Gαi2-KO constructs, with sgRNA2 or sgRNA3 (Fig. S2A), depleted Gαi2 protein expression in pHCC1 cells as well (Fig. S2B). The two potently decreased pHCC1 cell proliferation (EdU incorporation, Fig. S2C), in vitro cell migration (Fig. S2D) and invasion (Fig. S2E). These results supported that Gαi2 knockdown or KO potently inhibited pHCC1 cell cycle progression, proliferation, in vitro cell migration, and invasion.

Next, to the primary HCC cells derived from two other patients, pHCC2/pHCC3, and immortalized HepG2 cells, shGαi2-s1-expressing lentivirus was added. Puromycin was included to select stable cell colonies. As compared to HCC cells with sh-scr, *Gαi2* mRNA levels were remarkably decreased in shGαi2-s1-expresing HCC cells (Fig. 3H). shGαi2 failed to significantly alter *Gαi1* and *Gα3* mRNA expression in the primary and immortalized HCC cells (Fig. 3I). In the HCC cells, shRNA-induced silencing of Gαi2 robustly inhibited cell proliferation (Fig. 3J) and in vitro cell migration (Fig. 3K), which were separately tested by nuclear EdU staining (Fig. 3J) and “Transwell” (Fig. 3K) assays.

### Gαi2 depletion activates apoptosis in HCC cells

**Fig. 4:**
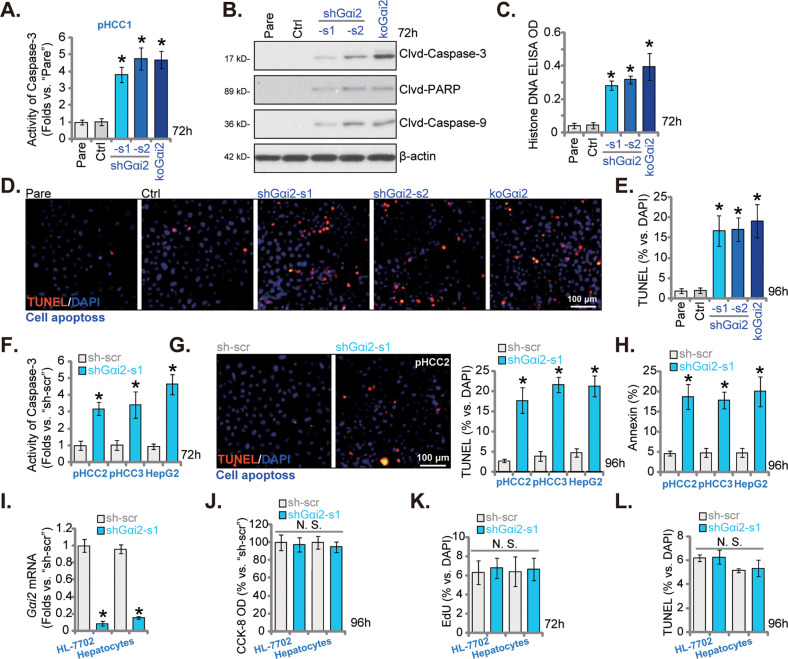
Gαi2 depletion activates apoptosis in HCC cells. The exact same amount of viable pHCC1 primary human HCC cells with applied genetic modifications on Gαi2, including shRNA-induced knockdown and dCas9/sgRNA1-induced knockout, were maintained in complete medium and cultivated for indicated time periods, the relative Caspase-3 activity (**A**), expression of the apoptosis-associated proteins (**B**) and Histone-bound DNA contents (**C**) were tested; Cell apoptosis was evaluated by measuring the percentages of TUNEL-positive nuclei (**D**, **E**). The control cells (“Ctrl”) were stably transduced with scramble control shRNA (sh-scr) plus lenti-CRISPR/dCas9-puro construct. The exact same amount of viable pHCC2 and pHCC3 primary cells or immortalized HepG2 cells, expressing the lentiviral shGαi2-s1or sh-scr, were cultured for indicated time periods, the relative Caspase-3 activity (**F**), the percentages of TUNEL-positive nuclei (% vs. DAPI) (**G**) and Annexin V-positive cells (**H**) were measured. Expression of Gαi2 in HL-7702 hepatocytes or the primary human adult hepatocytes (“Hepatocytes”) with shGαi2-s1or “sh-scr” was shown (**I**); The hepatocytes were further cultivated for indicated time periods, cell viability, proliferation and apoptosis were measured by CCK-8 (**J**), nuclear EdU staining (**K**) and TUNEL staining (**L**) assays, respectively, with results quantified. “Pare” stands for the parental control cells. Data were presented as mean ± standard deviation (SD, n = 5). *P < 0.05 versus “Pare”/“sh-scr” cells. “N. S.” stands for non-statistical difference (*P* > 0.05). Experiments were repeated three times with similar results obtained. Scale bar= 100 μm.

Since Gαi2 depletion caused HCC cell cycle arrested and hindered cell growth, we tested its effect on cell apoptosis. Caspase-3 activity was first examined and it was significantly increased in shGαi2-s1/2-expressing pHCC1 cells and koGαi2 (sgRNA1) pHCC1 cells (Fig. 4A). Moreover, cleavages of Caspase-3, Caspase-9 and poly (ADP-ribose) polymerase-1 (PARP) were increased in Gαi2-silenecd/-KO (sgRNA1) pHCC1 cells (Fig. 4B). The histone-bound DNA contents, evaluated by an ELISA assay, were increased in pHCC1 primary cancer cells with Gαi2 shRNA or the koGαi2 (sgRNA1) (Fig. 4C). Further studies revealed that Gαi2 silencing or KO (sgRNA1) activated apoptosis in pHCC1 cells and increased TUNEL-positive nuclei ratio (Fig. 4D, E).

In pHCC2 and pHCC3 primary HCC cells and immortalized HepG2 cells, shRNA-induced silencing of Gαi2, by shGαi2-s1 (see Fig. 3), similarly increased the Caspase-3 activity (Fig. 4F). Apoptosis was activated in Gαi2-silenced HCC cells. As the numbers of TUNEL-positive nuclei (Fig. 4G) and Annexin V-positive staining cells (Fig. 4H) were substantially increased following Gαi2 silencing in pHCC2/pHCC3 primary cells and HepG2 cells. These results clearly supported that Gαi2 depletion activated apoptosis in HCC cells. To the primary human adult hepatocytes and immortalized HL-7702 hepatocytes, shGαi2-s1-expressing lentivirus was added and stable hepatocytes established after selection. *Gαi2* mRNA levels were remarkably decreased in shGαi2-s1-expressing hepatocytes (Fig. 4I). Notably, shRNA-induced silencing of Gαi2 failed to result in viability reduction (Fig. 4J), proliferation inhibition (Fig. 4K) and apoptosis (Fig. 4L), which were measured by CCK-8 (Fig. 4J), nuclear EdU staining (Fig. 4K) and TUNEL staining (Fig. 4L) assays, respectively. These results supported the cancer cell-specific effect by Gαi2 depletion.

### Gαi2 depletion induces oxidative injury in HCC cells

**Fig. 5:**
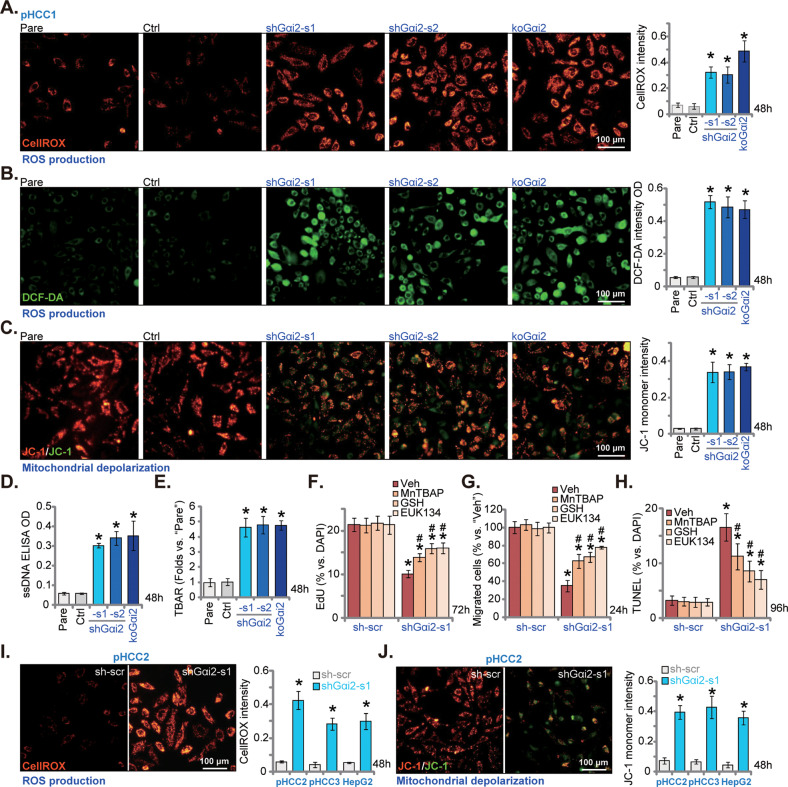
Gαi2 depletion induces oxidative injury in HCC cells. The exact same amount of viable pHCC1 primary human HCC cells with applied genetic modifications on Gαi2, including shRNA-induced knockdown and dCas9/sgRNA1-induced knockout, were maintained in complete medium and cultivated for 48 h, ROS production (by measuring CellROX/DCF-DA intensity, **A** and **B**), mitochondrial depolarization (by measuring JC-1 green monomers, **C**), DNA damage (by testing ssDNA contents, **D**) and lipid peroxidation (by examining TBAR intensity, **E**) were measured. The exact same amount of viable pHCC1 cells expressing shGαi2-s1 or sh-scr were treated with the antioxidant glutathione (GSH, 2 mM), EUK134 (25 μM), manganese tetrakis benzoic acid porphyrin (MnTBAP, 10 μM) or vehicle control (“Veh”) for indicated time periods, cell proliferation, migration and apoptosis were tested by nuclear EdU staining (**F**), “Transwell” (**G**) and nuclear TUNEL staining (**H**) assays, respectively. The exact same amount of viable pHCC2/pHCC3 primary cells or immortalized HepG2 cells, expressing shGαi2-s1 or sh-scr, were cultured for 48 h; ROS production and mitochondrial depolarization were tested by CellROX staining (**I**) and JC-1 staining (**J**) assays, respectively. Data were presented as mean ± standard deviation (SD, *n* = 5). *P < 0.05 versus “Pare”/“sh-scr” cells. ^#^*P* < 0.05 versus “Veh” treatment (**F**–**H**). Experiments were repeated three times with similar results obtained. Scale bar = 100 μm.

It has been shown that targeted inhibition of *Gαi2* in cardiomyocytes enhanced ischemic stress-induced oxidative [39]. Moreover, transcription factors binding to antioxidant response elements (ARE) could promote the transcriptional activation of Gαi2 [18]. These studies implied a potential role of Gαi2 in antioxidant response. We therefore analyzed whether Gαi2 depletion could provoke oxidative stress in HCC cells. As shown the CellROX red fluorescence intensity (Fig. 5A) and the DCF-DA green fluorescence intensity (Fig. 5B) were indeed increased in shGαi2-s1/2-expressing pHCC1 cells and koGαi2 (sgRNA1) pHCC1 cells. Moreover, Gαi2 silencing or KO (sgRNA1) induced mitochondrial depolarization by causing JC-1 green monomers accumulation (Fig. 5C) in pHCC1 cells. Moreover, Gαi2 depletion also resulted in significant DNA damage and increased single strand DNA (ssDNA) contents in pHCC1 cells (Fig. 5D). In addition, increased TBAR intensity supported lipid peroxidation in pHCC1 cells with Gαi2 depletion (Fig. 5E). These results clearly supported that Gαi2 depletion induced oxidative injury in pHCC1 cells.

Next, we showed that shGαi2-s1-induced proliferation arrest, or EdU ratio reduction, was largely inhibited by different antioxidants, including the superoxide dismutase (SOD) mimetic MnTBAP [40, 41], reduced glutathione and the SOD and catalase mimics EUK134 [42, 43] (Fig. 5F). Moreover, Gαi2 silencing-induced pHCC1 cell migration inhibition was ameliorated following treatment with the antioxidants (Fig. 5G). In addition, the applied antioxidants attenuated shGαi2-s1-induced pHCC1 cell apoptosis, which was tested by nuclear TUNEL staining assays (Fig. 5H). In pHCC2/pHCC3 primary cells and HepG2 cells, Gαi2 silencing by shGαi2-s1 similarly induced oxidative injury and increased CellROX red fluorescence intensity (Fig. 5I). Furthermore, the accumulation of JC-1 green monomers, supporting mitochondrial depolarization, was detected in Gαi2-silenced primary and immortalized HCC cells (Fig. 5J). These results further supported that Gαi2 depletion provoked oxidative injury in primary and established HCC cells.

### Ectopic overexpression of Gαi2 further promotes HCC cell proliferation and migration

**Fig. 6:**
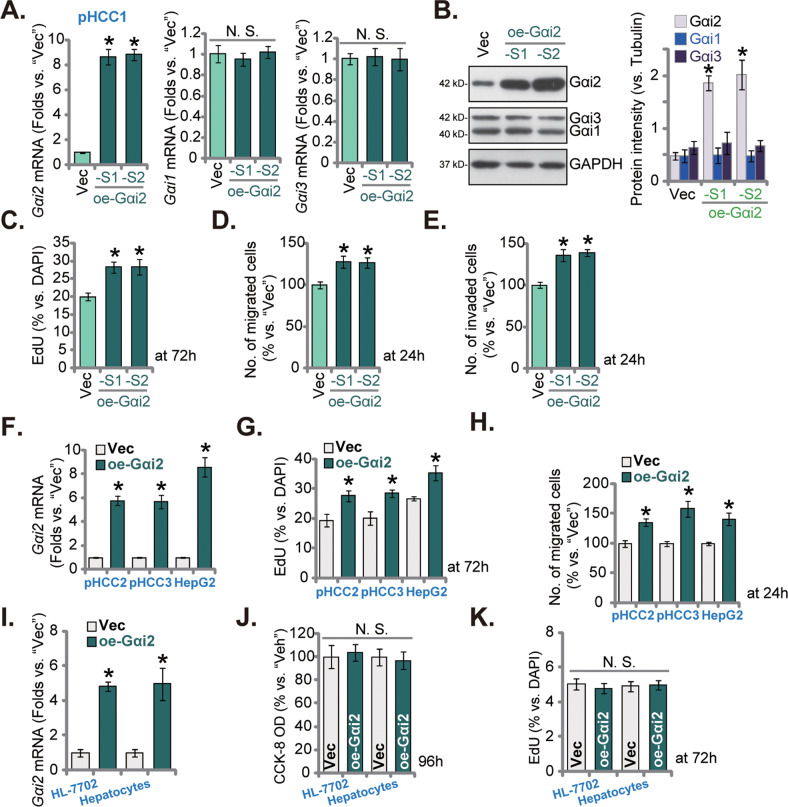
Ectopic overexpression of Gαi2 further promotes HCC cell proliferation and migration. The two stable pHCC1 cell selections, oe-Gαi2-S1 and oe-Gαi2-S2, with the lentiviral Gαi2-overexpressing construct were formed following selection; Control cells were stably transduced with the empty vector (“Vec”); Expression of *Gαi1/Gαi2/Gαi3* mRNAs and listed proteins was tested by qRT-PCR (**A**) and Western blotting (**B**). The exact same amount of viable pHCC1 cells were cultivated for indicated time periods, cell proliferation, in vitro cell migration and invasion were examined by nuclear EdU staining (**C**), “Transwell” (**D**) and “Matrigel Transwell” (**E**) assays, respectively, with results quantified. The exact same amount of viable pHCC2 and pHCC3 primary cells, immortalized HepG2 cells, HL-7702 hepatocytes or the primary human adult hepatocytes (“Hepatocytes”), expressing the lentiviral Gαi2-overexpressing construct (“oe-Gαi2”) or the empty vector (“Vec”) were cultured, and expression of *Gαi1/Gαi2/Gαi3* mRNAs tested (**F**, **I**). Cells were cultivated for indicated time periods, cell proliferation (**G**, **K**), in vitro cell migration (**H**) and viability (**J**) were tested similarly. Data were presented as mean ± standard deviation (SD, *n* = 5). *P < 0.05 versus “Vec” cells. “N. S.” stands for non-statistical difference (*P* > 0.05). Experiments were repeated three times with similar results obtained.

To further support the role of Gαi2 in HCC, a lentiviral construct encoding *Gαi2* cDNA sequence was transduced to pHCC1 cells. After treatment with puromycin-containing medium, two stable pHCC1 cell selections, oe-Gαi2-S1 and oe-Gαi2-S2, were formed. As shown, *Gαi2* mRNA expression increased over 8-9 folds in oe-Gαi2-expressing pHCC1 cells (Fig. 6A), and *Gαi1* and *Gαi3* mRNA unchanged (Fig. 6A). Gαi2 protein upregulation was observed in oe-Gαi2-S1 and oe-Gαi2-S2 pHCC1 cells as well (Fig. 6B), with Gαi1/Gαi3 protein levels not altered (Fig. 6B). With Gαi2 overexpression, the EdU-positive nuclei ratio was remarkably increased in pHCC1 cells (Fig. 6C), supporting that Gαi2 overexpression further promoted HCC cell proliferation. Moreover, in vitro pHCC1 cell migration and invasion were accelerated after Gαi2 overexpression (see quantified results in Fig. 6D and E). Therefore, ectopic overexpression of Gαi2 exerted pro-cancerous activity in pHCC1 cells.

The same lentiviral Gαi2-overexpresing construct was stably transduced to pHCC2/pHCC3 primary cells and HepG2 cells, causing robust upregulation of *Gαi2* mRNA (Fig. 6F). In the primary and immortalized HCC cells, Gαi2 overexpression enhanced nuclear EdU incorporation and augmented cell proliferation (Fig. 6G). In vitro cell migration, examined by “Transwell” assays, was also accelerated following Gαi2 overexpression (Fig. 6H). However in the primary human adult hepatocytes and HL-7702 hepatocytes, ectopic *Gαi2* overexpression, using the same lentiviral construct (Fig. 6I), failed to enhance cell viability (CCK-8 OD, Fig. 6J) and proliferation (EdU incorporation, Fig. 6K).

### The binding between the transcription factor EGR1 and *Gαi2* promoter is increased in HCC tissues and cells

**Fig. 7:**
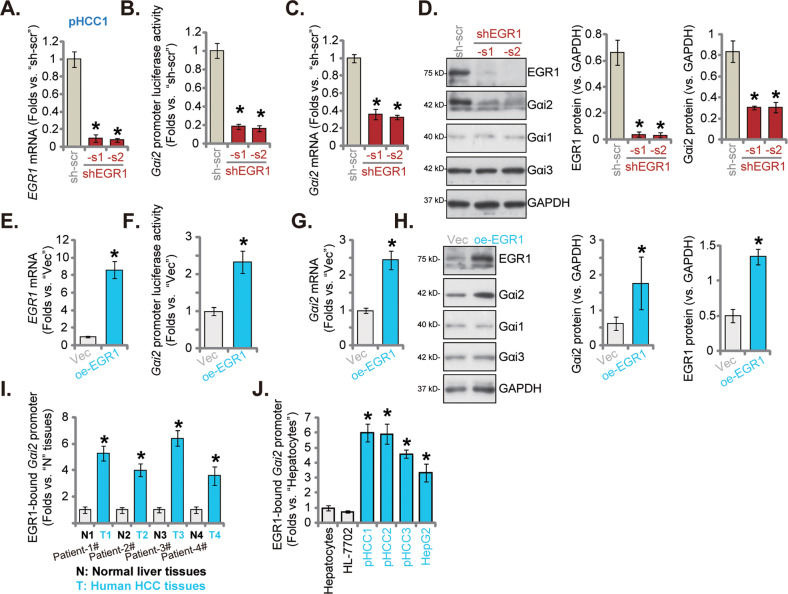
The binding between the transcription factor EGR1 and *Gαi2* promoter is increased in HCC tissues and cells. The stable pHCC1 cells expressing the lentiviral EGR1 shRNA (shEGR1-s1 or shEGR1-s2, with non-overlapping sequences), the sh-scr (**A**–**D**), the lentiviral EGR1-expresing construct (“oe-EGR1”) or the empty vector (“Vec”) (**E**–**H**) were formed and expression of listed genes and proteins was shown (**A**, **C**, **D**, **E**, **G** and **H**). The *Gαi2* promoter luciferase activity was analyzed and results were quantified (**B**, **F**). Chromosome IP (ChIP) showed the relative levels of the Gαi2 promoter binding to the EGR1 protein in the listed human tissues (**I**) and cells (**J**). The data were presented as mean ± standard deviation (SD). *P < 0.05 *vs*. “sh-scr”/“Vec”/“N” tissues/“Hepatocytes”. The in vitro experiments were repeated five times with similar results obtained.

The underlying mechanism of Gαi2 upregulation in HCC was also examined. Since both *Gαi2* mRNA and protein levels are elevated in HCC tissues and cells, we proposed that there could be a translational mechanism. Kinane et al. have reported that EGR1 could be an important transcription factor for Gαi2 in human cells [36]. Therefore, two different lentiviral shRNAs against human *EGR1*, shEGR1-s1 and shEGR1-s2 (containing non-overlapping sequences), were individually transduced to pHCC1 cells. Stable cells were formed following selection. The applied shRNAs resulted in remarkable *EGR1* mRNA (Fig. 7A). Significantly, the *Gαi2* promoter luciferase activity was significantly decreased following EGR1 silencing in pHCC1 cells (Fig. 7B). As a result, *Gαi2* mRNA was downregulated in EGR1-silenced pHCC1 cells (Fig. 7C). Moreover, protein expression of Gαi2, but not Gαi1 and Gαi3, was significantly decreased in EGR1-silenced pHCC1 cells (Fig. 7D), where EGR1 protein silencing was observed (Fig. 7D).

In contrast, the lentiviral particles encoding the EGR1-expresing construct were added to pHCC1 cells, stable cells (“oeEGR1”) were formed following puromycin selection. When compared to the vector control cells (“Vec”), *EGR1* mRNA expression (Fig. 7E) and the *Gαi2* promoter luciferase activity (Fig. 7F) were significantly elevated in oeEGR1 pHCC1 cells. *Gαi2* mRNA (Fig. 7G), Gαi2 protein (Fig. 7H), and EGR1 protein (Fig. 7H) upregulation was detected in EGR1-overexpressed pHCC1 cells as well. Gαi1 and Gαi3 protein levels were again unchanged (Fig. 7H). These results supported that EGR1 could be an important transcription factor of Gαi2 in HCC cells.

Importantly, ChIP assay results revealed that the binding between the EGR1 protein and *Gαi2* DNA promoter sequence was significantly increased in HCC tumor tissues of four different primary HCC patients (Patient-1#/-2#/-3#/-4#) (Fig. 7I). Where the EGR1 protein-*Gαi2* promoter binding was relative low in adjacent normal liver tissues (Fig. 7J). The binding affinity between the transcription factor EGR1 and *Gαi2* promoter was also significantly increased in primary (pHCC1/ pHCC-2/ pHCC-3) and HepG2 HCC cells, when compared to the low binding between the two in primary human hepatocytes and immortalized HL-7702 cells (Fig. 7I). Thus, the increased binding between the transcription factor EGR1 and *Gαi2* promoter could be the primary mechanism of Gαi2 upregulation in HCC.

### Gαi2 shRNA inhibits HCC xenograft growth in nude mice

**Fig. 8:**
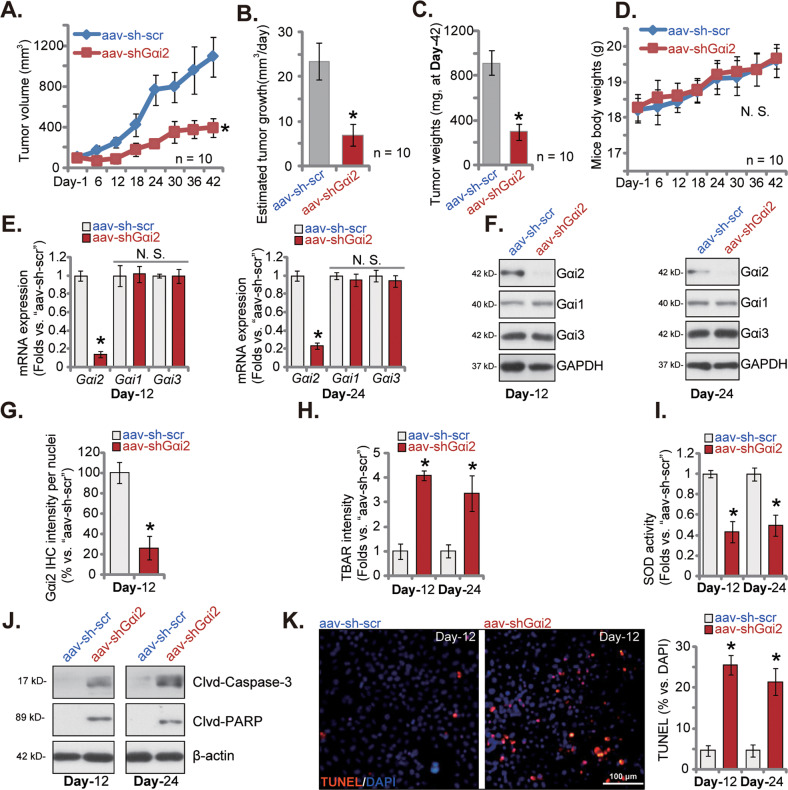
Gαi2 silencing suppresses pHCC1 cell xenograft growth in nude mice. The nude mice bearing subcutaneous pHCC1 xenografts were subject to intratumoral injection of shGαi2-s1-expressing adeno-associated virus (“aav-shGαi2”) or the scramble control shRNA adeno-associated virus (“aav-sh-scr”), and ten mice in each group (*n* = 10). Virus was injected every 48 h for five rounds. The pHCC1 cell xenografts’ volumes (**A**) and animal body weights (**D**) were recorded every six days. The estimated daily tumor growth (in mm^3^ per day) was calculated (**B**). At Day-42, all pHCC1 xenografts were carefully isolated and weighted (**C**). The listed pHCC1 xenografts were homogenized, and listed genes and proteins in the tissue lysates tested (**E**, **F** and **J**). The TBAR intensity (**H**) and the SOD activity (**I**) in xenograft tissue lysates were examined. The relative Gαi2 IHC quantification results in pHCC1 xenograft slides were shown (**G**). The representative TUNEL fluorescence images in the described pHCC1 xenograft slides were shown (**K**). The data were presented as mean ± standard deviation (SD). *P < 0.05 versus “aav-sh-scr” group. “N. S.” stands for non-statistical difference (*P* > 0.05). Scale bar= 100 μm.

We explored the potential role of Gαi2 on HCC cell growth in vivo. The mice xenograft assay was carried out. A significant number pHCC1 (six million cells per mouse) were *s.c*. injected to the flanks of nude mice. pHCC1 xenografts were then formed after three weeks (labeled as experimental “Day-1”). Thereafter, the nude mice bearing the pHCC1 xenografts were assigned randomly into two groups, with ten mice of each group (n = 10). The first group received intratumoral injection of Gαi2 shRNA (“shGαi2-s1”)-expressing adeno-associated virus (“aav-shGαi2”); Whereas the other group mice were intratumorally injected with scramble control shRNA adeno-associated virus (“aav-sh-scr”). The virus was injected every 48 h for a total of five rounds. As shown, aav-shGαi2 injection remarkably hindered pHCC1 xenograft growth in nude mice (Fig. 8A). The volumes of aav-shGαi2 group pHCC1 xenografts were remarkably lower than those of aav-sh-scr xenografts (Fig. 8A). When calculating daily tumor growth, in mm^3^ per day, we further showed that pHCC1 xenograft growth was robustly suppressed after aav-shGαi2 injection (Fig. 8B). All pHCC1 xenografts were separately carefully at Day-42. The aav-shGαi2 pHCC1 xenografts were significantly lighter than aav-sh-scr xenografts (Fig. 8C). The body weights of the nude mice, as shown in Fig. 8D, were indifferent between aav-shGαi2 mice and aav-sh-scr mice. These results showed that Gαi2 shRNA virus injection robustly inhibited HCC xenograft growth in nude mice.

Next, at Day-12 and Day-24, one pHCC1 xenograft in each group was isolated and total four xenografts were examined. As shown, *Gαi2* mRNA levels were substantially decreased in aav-shGαi2-expressing pHCC1 xenografts, where *Gαi1* and *Gαi3* mRNA levels were unchanged (Fig. 8E). Gαi2 protein downregulation was also detected in pHCC1 xenografts after intratumoral injection of Gαi2 shRNA aav (Fig. 8F), and Gαi1 and Gαi3 protein expression unaffected (Fig. 8F). The quantified IHC results in pHCC1 xenograft slides further supported Gαi2 *protein* silencing in aav-shGαi2-injected pHCC1 xenografts (Fig. 8G). Further analyzing xenograft tissues revealed that the TBAR activity was robustly enhanced in the Gαi2-silenced pHCC1 xenograft tissues (Fig. 8H). Further supporting oxidative injury, we found that SOD activity was decreased in aav-shGαi2 pHCC1 xenograft tissues (Fig. 8I). In Fig. 8J we found that levels of cleaved-Caspase-3 and cleaved-PARP were increased in pHCC1 xenograft tissues with Gαi2 silencing. Moreover, the TUNEL fluorescence staining in pHCC1 xenograft slides supported apoptosis activation in Gαi2-silenced pHCC1 xenografts, as the TUNEL-positive nuclei ratio was robustly increased (Fig. 8K). These results together showed that Gαi2 silencing-induced oxidative injury and apoptosis in pHCC1 xenograft tissues.

### Gαi2 KO hinders HCC cell growth in vivo

**Fig. 9:**
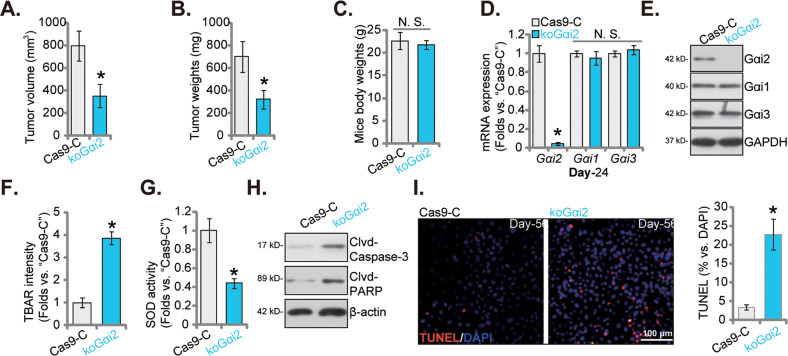
Gαi2 KO hinders HCC cell growth in vivo. pHCC1 cells with the Gαi2 sgRNA1-expressing lenti-CRISPR/dCas9-KO-puro construct (“koGαi2”) or the lenti-CRISPR/dCas9-KO-puro construct (“Cas9-C”) were *s.c*. injected to flanks of the nude mice at 1 × 10 ^7^ cells per xenograft. There were six mice in each group. After seven weeks, the xenografts were isolated, tumor volumes (**A**), tumor weights (**B**) and animal body weights (**C**) were measured. pHCC1 cell xenografts were homogenized, and listed genes and proteins in the tissue lysates tested (**D**, **E,** and **H**). The TBAR intensity (**F**) and the SOD activity (**G**) in the xenograft tissues were examined. The representative TUNEL fluorescence images in the described pHCC1 xenograft slides were presented (**I**). The data were presented as mean ± standard deviation (SD, *n* = 6). *P < 0.05 versus “Cas9-C” group. “N. S.” stands for non-statistical difference (*P* > 0.05). Scale bar= 100 μm.

To further support the role of Gαi2 on HCC cell growth in vivo, koGαi2 (sgRNA1) pHCC1 cells and control cells with the lenti-CRISPR/dCas9-KO-puro construct (“Cas9-C”) were *s.c*. injected to flanks of the nude mice. After seven weeks, the xenografts were isolated and measured. As shown, koGαi2 pHCC1 xenografts were much smaller (Fig. 9A) and lighter (Fig. 9B) than Cas9-C pHCC1 xenografts. The mice body weights were indifferent between koGαi2 and Cas9-C mice (Fig. 9C). *Gαi2* mRNA and protein expression was depleted in koGαi2 pHCC1 xenografts (Fig. 9D, E), and *Gαi1* and *Gαi3* mRNA and protein expression unchanged (Fig. 9D, E). TBAR intensity was increased in koGαi2 pHCC1 xenograft tissues (Fig. 9F), and SOD activity decreased (Fig. 9G). Increased Caspase-3-PARP cleavages (Fig. 9H) and nuclear TUNEL staining (Fig. 9I) in koGαi2 pHCC1 xenografts supported apoptosis activation. These results together supported that Gαi2 KO inhibited pHCC1 xenograft growth in nude mice, and inducing oxidative injury and apoptosis activation.

## Discussion

HCC is currently the third leading cause of cancer-related human death around the world with its incidence rising [3, 4]. Radiofrequency/microwave ablation, liver resection or liver transplantation are the potential curative therapies for early stage HCC [3, 4]. For the advanced HCC patients, the molecularly targeted therapies are important. Dysregulation of multiple signaling cascades, including Wnt/β-catenin, p53, Akt-mTOR, VEGFR and EGFR, are often detected in HCC, and playing significant roles in carcinogenesis and cancer progression [9–12]. Clinical trials are underway testing molecular therapies against key signaling proteins in these cascades [9–12].

Our group and others have identified two other components of Gαi family proteins, Gαi1 and Gαi3, are possible key oncogenic targets for human cancer [15–19]. Gαi1 and Gαi3 are upregulated in human glioma tissues and cells, mediating Akt-mTOR activation to promote glioma cell growth in vitro and in vivo [15–17]. In osteosarcoma, overexpressed Gαi3 associated with multiple RTKs to mediate downstream Akt signaling activation, driving osteosarcoma cell growth [19]. Moreover, upregulated Gαi3 is a promising novel oncotarget of cervical cancer [18]. Gαi3 silencing or KO potently inhibited cervical cancer cell growth in vitro and in vivo [18].

Nguyen et al. reported that Gαi1 protein expression was significantly upregulated in both human alcoholic steatohepatitis (ASH) tissues and non-alcoholic steatohepatitis (NASH) tissues, being more significant in ASH tissues [44]. Interestingly, Yao et al. have shown that Gαi1 is downregulated in HCC and functions as a potential cancer-suppressing protein [45]. Specifically, Gαi1 expression inhibited HCC cell migration and invasion [45]. Chen et al. reported that *Gαi3* expression is decreased in human HCC tissues and its low expression correlates with poor prognosis in HCC patients [46]. Zhang et al. further showed that Gαi3 protein expression is downregulated in human HCC tissues and Gαi3 inhibited HCC cell migration/invasion [47].

The results of this study implied that Gαi2 could be a promising therapeutic target and novel diagnosis marker of HCC. TCGA-LIHC database shows that the number *Gαi2* transcripts in HCC tissues is significantly higher than that in the normal liver tissues. Moreover, *Gαi2* overexpression in HCC tissues correlates with poor prognosis of the patients. *Gαi2* mRNA and protein expression is also elevated in local HCC tissues and different human HCC cells. In primary HCC cells and immortalized HepG2 cells, Gαi2 shRNA or KO largely suppressed cell growth and migration, while inducing cell cycle arrest and caspase-apoptosis activation. Depletion of Gαi2 however failed to significantly inhibit viability and proliferation of normal hepatocytes. In vivo, intratumoral injection of Gαi2 shRNA AAV largely hindered growth of pHCC1 xenografts in nude mice. Moreover, Gαi2-KO pHCC1 xenografts growth was significantly slowed in nude mice. Thus, targeting Gαi2 could be a promising molecularly targeted therapy for HCC.

A fine strategy to inhibit HCC cell growth and to activate apoptosis is through inducing ROS production and oxidative stress. Metabolic activation of PCK1 (phosphoenolpyruvate carboxykinase 1) promoted energy crisis, oxidative stress and apoptosis in HCC cells and inhibited HCC cell growth in vitro and in vivo [48]. Wang et al. revealed that CDCA2 (cell division cycle associated 2) promoted HCC cell growth and inhibited apoptosis possibly by activating NRF2 signaling axis and inhibited oxidative stress [49]. Artesunate and sorafenib synergistically induced oxidative stress, lipid peroxidation and ferroptosis to inhibit HCC cell growth [50]. In the present study, we found that Gαi2 silencing or KO-induced ROS production and oxidative injury in primary and immortalized HCC cells. Whereas different antioxidants ameliorated Gαi2-shRNA-induced anti-HCC cell activity. Moreover, oxidative injury and apoptosis were detected in Gαi2-silenced or Gαi2-KO pHCC1 xenografts. Therefore, Gαi2-driven HCC cell growth could be due to, at least in part, by suppressing oxidative injury and apoptosis.

EGR1 could play an important role in tumorigenesis and cancer progression by transcriptional activation its targets. Li et al. showed that EGR1 promoted prostate cancer metastasis by inducing the expression of angiogenic and osteoclastogenic factors [50]. Ma et al. found that EGR1 activated the transcription of long non-coding RNA linc01503 to promote cell cycle progression and tumorigenesis of gastric cancer [51]. Liu et al. have shown that EGR1-activated transcription of LncRNA HNF1A-AS1 promoted gastric cancer cell cycle progression [52]. Here we found that EGR1-activated transcription activation of *Gαi2* could be an important mechanism of Gαi2 overexpression in HCC. In pHCC1 cells, *Gαi2* mRNA and protein expression was significantly decreased following EGR1 silencing, but was elevated after ectopic EGR1 overexpression. ChIP assay results showed that the binding between EGR1 and *Gαi2* DNA promoter is increased in HCC tissues and cells. Thus, EGR1-induced transcription activation of *Gαi2* promoted HCC cell growth in vitro and in vivo.

## Conclusion

Overexpressed Gαi2 is required for HCC cell growth in vitro and in vivo, representing as a novel and promising diagnosis marker and therapeutic target of HCC.

## Supplementary information

Authorship change form

Figure S1

aj-checklist form

Author contribution form

## Data Availability

All data are available upon request.
