# Supplementary material for: Identification of G protein subunit alpha i2 as a promising therapeutic target of hepatocellular carcinoma
Source: Cell Death Dis. 2023 Feb 20;14(2):143. doi: 10.1038/s41419-023-05675-6 (PMC9941495; doi:10.1038/s41419-023-05675-6)
Supplement: Supplementary file 2 — Figure S1 [file 41419_2023_5675_MOESM2_ESM.pdf]

Figure S1. The uncropped blotting images

Figure 2.

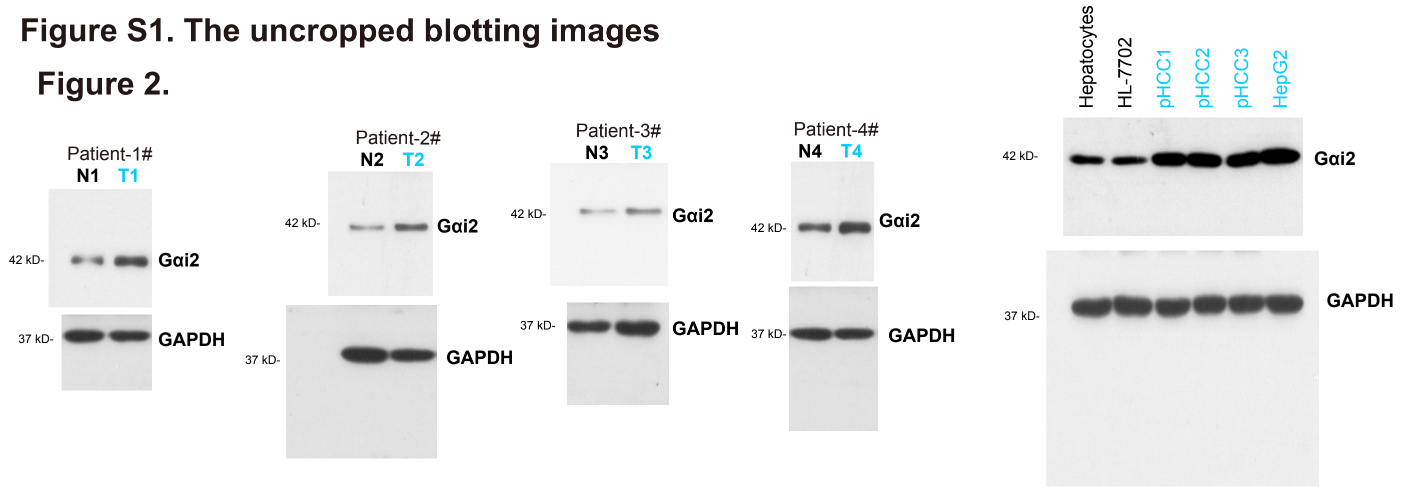

Figure 3.

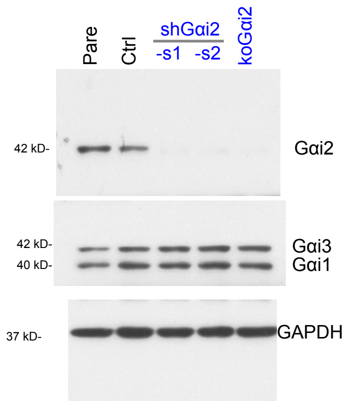

Figure 4.

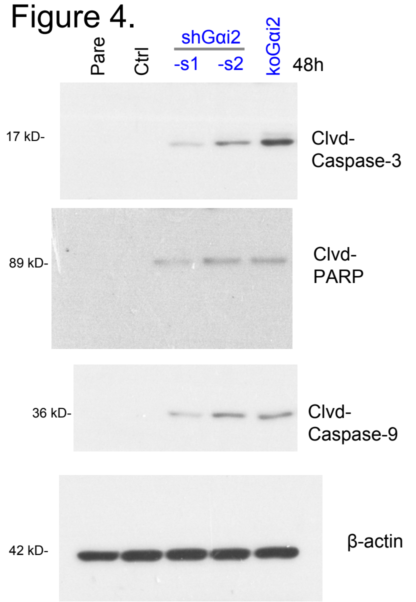

Figure 6.

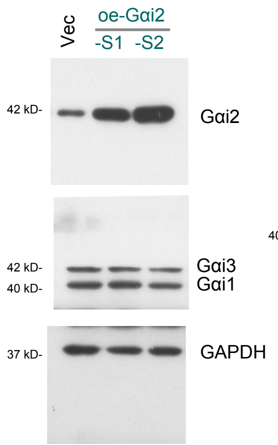

Figure 9.

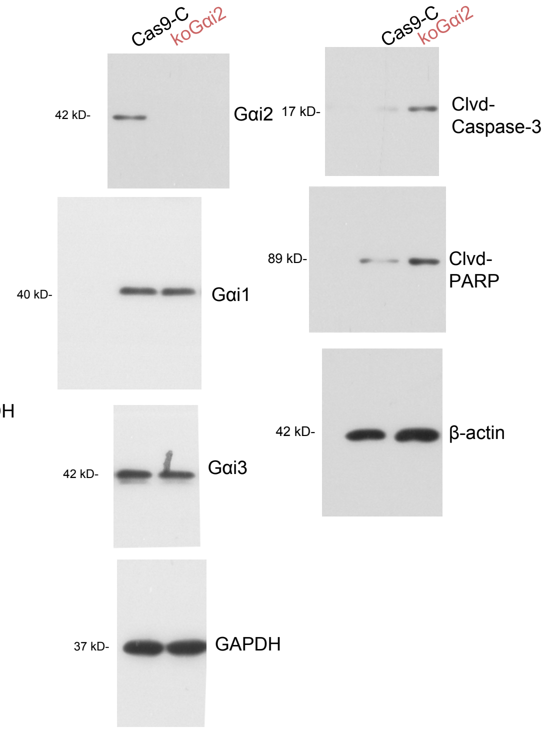

Figure 7.

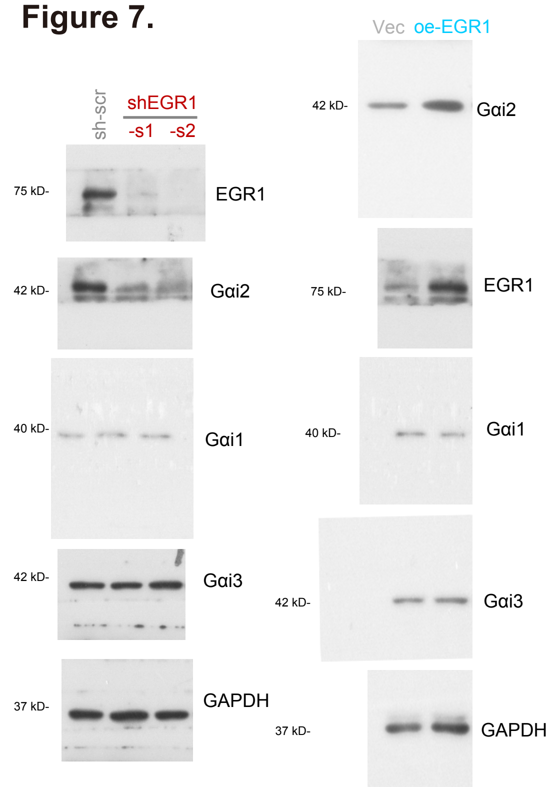

Figure 8.

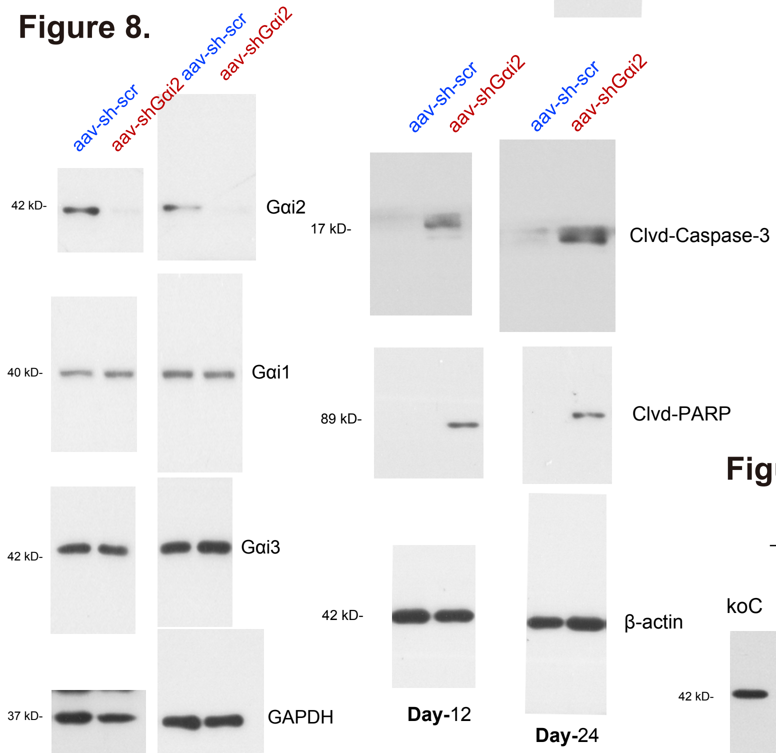

Figure S2.

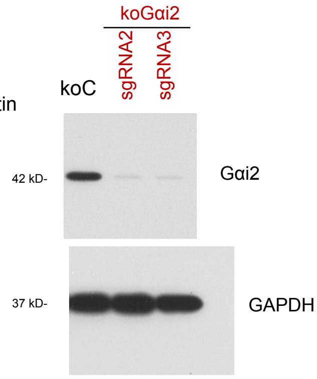

Figure S2.

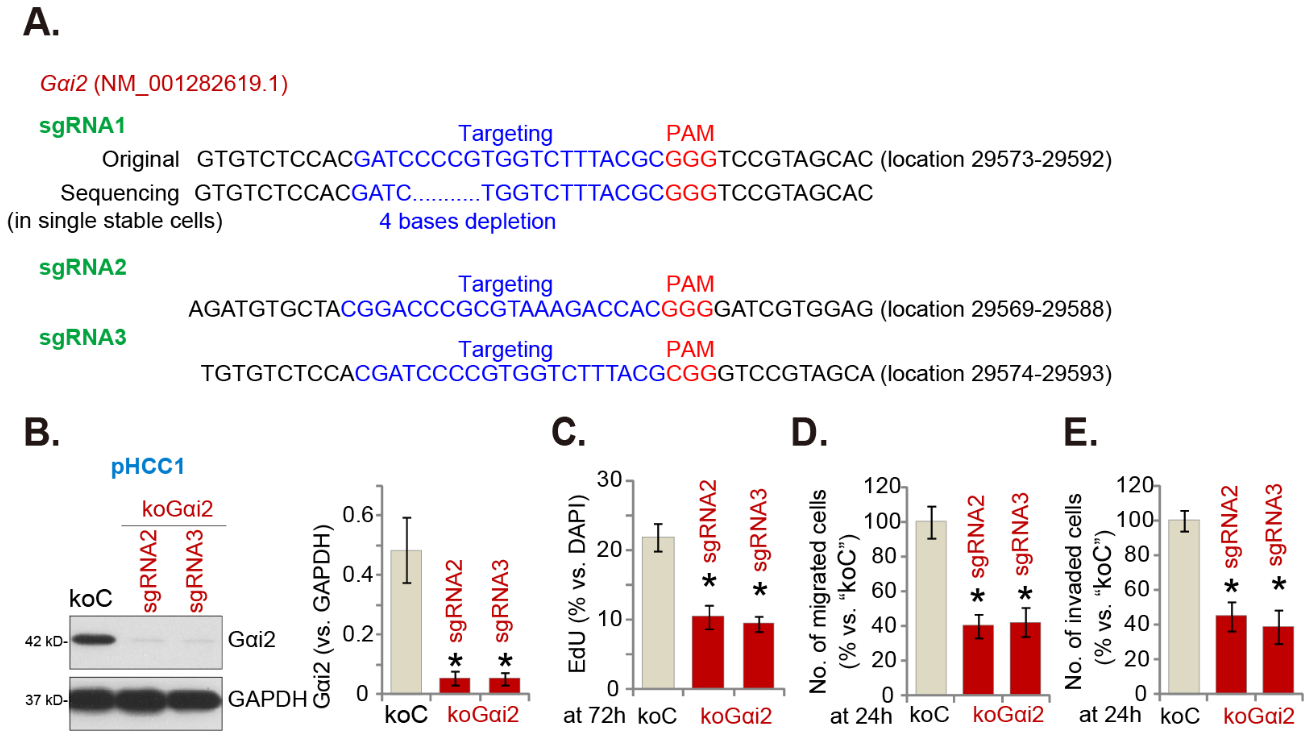

Figure S2: The small guide (sgRNA) targeting DNA sequence, PAM sequence, and sequencing verification in the stable cells for the three CRISPR/dCas-9-Gα i2-KO constructs ("koGαi2-sgRNA1-2/-3") were listed (**A**). The exact same amount of viable pHCC1 cells, expressing dCas9 and the CRISPR/dCas-9-Gα i2-KO construct containing sgRNA against Gα i2 ("koGαi2-sgRNA2/3") or the lenti-CRISPR/dCas9-puro construct ("koC"), were maintained in complete medium, expression of Gα i2 and GAPDH was shown (**B**); Cells were further cultivated for applied time periods, cell proliferation, *in vitro* cell migration and invasion were examined by the nuclear EdU staining (**C**), "Transwell" (**D**) and "Matrigel Transwell" (**E**) assays, respectively, with results quantified. Data were presented as mean ± standard deviation (SD, n=5). * ***P*** < 0.05 versus "koC" cells. Experiments were repeated three times with similar results obtained.
