# Supplementary material for: Identification of G protein subunit alpha i2 as a promising therapeutic target of hepatocellular carcinoma
Source: Cell Death Dis. 2023 Feb 20;14(2):143. doi: 10.1038/s41419-023-05675-6 (PMC9941495; doi:10.1038/s41419-023-05675-6)
Supplement: Supplementary file 4 — Author contribution form [file 41419_2023_5675_MOESM4_ESM.pdf]

DECLARATION OF CONTRIBUTIONS TO ARTICLE

ADMC

Manuscript Number: CDDIS-22-4701R

Journal Name: *Cell Death & Disease* (the 'Journal')

Proposed Title of the Contribution:

Identification of G protein subunit alpha i2 as a promising therapeutic target of hepatocellular carcinoma (the 'Contribution)

Author(s):

Minbin Chen, Zhifei Li, Chengtao Gu, Hao Zheng, Yan Chen, and Long Cheng (the 'Authors')

For all *CDDis* articles, each person named as an author in the published version must be able to show he or she has contributed substantially to the article.

Authorship credit should be based on 1) substantial contributions to conception and design, acquisition of data, or analysis and interpretation of data; 2) drafting the article or revising it critically for important intellectual content; and 3) final approval of the version to be published. Authors should meet conditions 1, 2 and 3.

Any person who cannot be shown to have made a substantial contribution to the article cannot be listed as an author in the final version. The name of any person who is deemed to have made a minor contribution can, however, appear in the Acknowledgments section of the article.

Please complete the table below to indicate the contributions of all named authors to the manuscript.

Author Full Name:	Specification of Contribution to the Manuscript:
Minbin Chen	Culture primary HCC cells and normal hepatocytes, testing testing signalings, performed the in vivo studies, drafting the article and revising it, with final approval of the version submitted to the journal.
Zhifei Li	Culture primary HCC cells and normal hepatocytes, testing testing signalings, performed the in vivo studies, drafting the article and revising it, with final approval of the version submitted to the journal.
Chengtao Gu	Culture primary HCC cells and normal hepatocytes, testing testing signalings, performed the in vivo studies, drafting the article and revising it, with final approval of the version submitted to the journal.
Hao Zheng	Culture primary HCC cells and normal hepatocytes, testing testing signalings, performed the in vivo studies, drafting the article and revising it, with final approval of the version submitted to the journal.
Yan Chen	Study conception and design, and data analysis, Figure organization, involved in drafting the article and revising it critically for important intellectual content, and with final approval of the version submitted to the journal.
Long Cheng	Study conception and design, and data analysis, Figure organization, involved in drafting the article and revising it critically for important intellectual content, and with final approval of the version submitted to the journal.

Please complete the table below to indicate the contributions of all named authors to the figures.

Figure 1:

MC, ZL, CG, HZ and LC

Figure 2:

MC, ZL, CG, HZ and LC

Figure 3:

MC, ZL, CG, HZ, YC, and LC

Figure 4:

MC, ZL, CG, HZ and LC

Figure 5:

MC, ZL, CG, HZ, YC, and LC

Figure 6:

MC, ZL, CG, HZ, YC, and LC
Figure 7-9, MC, ZL, CG, HZ, YC, and LC

Signed for and on behalf of the Author(s):	Print Name:	Date:
Long Cheng	Long Cheng	02/03/2023
